# Patch area and uniform sampling on the surface of any ellipsoid

**DOI:** 10.1007/s11075-023-01628-4

**Published:** 2023-08-14

**Authors:** Callum Robert Marples, Philip Michael Williams

**Affiliations:** Molecular Therapeutics and Formulation, School of Pharmacy, University of Nottingham, Nottingham, NG7 2RD UK

**Keywords:** Ellipsoid, Surface area, Random sampling, 53A05, 53-08, 65D18

## Abstract

Algorithms for generating uniform random points on a triaxial ellipsoid are non-trivial to verify because of the non-analytical form of the surface area. In this paper, a formula for the surface area of an ellipsoidal patch is derived in the form of a one-dimensional numerical integration problem, where the integrand is expressed using elliptic integrals. In addition, analytical formulae were obtained for the special case of a spheroid. The triaxial ellipsoid formula was used to calculate patch areas to investigate a set of surface sampling algorithms. Particular attention was paid to the efficiency of these methods. The results of this investigation show that the most efficient algorithm depends on the required coordinate system. For Cartesian coordinates, the gradient rejection sampling algorithm of Chen and Glotzer is best suited to this task, when paired with Marsaglia’s method for generating points on a unit sphere. For outputs in polar coordinates, it was found that a surface area rejection sampler is preferable.

## Introduction

Generating uniform random samples of points on surfaces finds many applications. Some examples include: solving radiation transport problems in medical physics [1], quantifying errors in brain image analysis techniques [2], modelling oxygen production of trees in forests [3], simulating the effect of background radiation on detector surfaces [4], statistical goodness-of-fit testing [5], solving problems in development of visualisation software [6] and testing robot motion planning algorithms [7]. The particular problem of sampling the surface of an ellipsoid has been of interest for modelling: dose-rate distributions of iodine-125 for radiation therapy [1], prolate virus capsid formation [8], protein coats of bacterial spores [9], and impacts on solar system objects of unusual shape (such as the bilobate Kuiper belt object, Arrokoth) [10].

Many published studies consider sampling of arbitrary surfaces [1, 3–6, 11], as well as the relatively simple example of the sphere [12–15]. However, there is relatively little published material that considers the ellipsoidal case. Williamson provided a method for the ellipsoid surface based on rejection sampling [1]. Chen and Glotzer improved upon this method, giving a proof and numerical verification for the case of a prolate spheroid [8]. While this method is just as valid for any other kind of ellipsoid, verifying the uniformity of a sample in the triaxial case is a highly non-trivial task because the surface area cannot be expressed as an analytical function.

An explicit expression for the surface area of the entirety of a general ellipsoid was first derived in 1825 by Legendre in terms of incomplete elliptic integrals of the first and second kinds (See Reference [16] for a historical review). Many studies have been undertaken regarding exact and approximate expressions for the surface area of the entire ellipsoid [16–24]. However, only one of these considers the problem of finding the area of a subset of the ellipsoid surface [17]. That work involved cutting the ellipsoid into two segments using an intersecting plane and finding the area of those segments.

In this study, the surface area of interest is that of a patch bounded by limits given in (scaled) spherical polar coordinates, ($\theta$, $\phi$), defined such that

1 $$\begin{aligned} x&= a\sin \theta \cos \phi \, , \end{aligned}$$

2 $$\begin{aligned} y&= b\sin \theta \sin \phi \, ,\end{aligned}$$

3 $$\begin{aligned} z&= c\cos \theta \, , \end{aligned}$$

where $\theta \in [0,\pi ]$ is an angle measured from the *c*-axis and $\phi \in [0,2\pi )$ is an azimuth angle defined in the *x*-*y* plane. It is assumed in this work that $a \ge b \ge c$. The sole exception to this is a prolate spheroid, for which it is assumed that $a = b < c$.

**Fig. 1:**
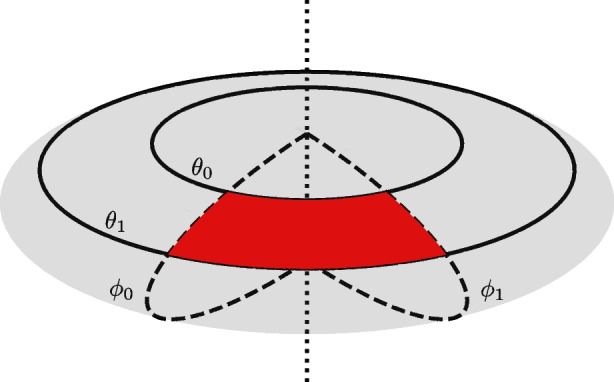
A patch on the surface of an ellipsoid. For limits $\theta _0$, $\theta _1$, $\phi _0$ and $\phi _1$, one can determine curves of constant $\theta$ (solid) and of constant $\phi$ (dashed). The patch (red) is defined as the area enclosed by the four curves. The dotted vertical line indicates the z-axis (from which $\theta$ is measured)

Figure 1 shows how an ellipsoidal patch is defined using limits $\theta _0$, $\theta _1$, $\phi _0$ and $\phi _1$. The patch is an ‘ellipsoidal rectangle’ with vertices $(\theta _0, \phi _0), (\theta _0, \phi _1), (\theta _1, \phi _0)$ and $(\theta _1, \phi _1)$. When a vertex is one of the two poles in $\theta$ (with $\theta = 0$ or $\pi$), the patch is no longer ‘rectangular’, but is now the cap of the ellipsoid (with no divisions in $\phi$).

In Sect. 2, an expression for the surface area of an ellipsoidal patch is derived and a means of numerically evaluating this expression is outlined. Given the surface areas of ellipsoidal patches, uniformity of ellipsoid surface sampling algorithms can be studied. In Sect. 3, measures of speed as well as statistics describing uniformity are discussed. These measures are applied in Sect. 4 to a selection of sampling algorithms on a spherical surface. In Sect. 5, some rejection sampling algorithms for the ellipsoid surface are outlined (some requiring sphere samplers to generate trial points). A similar analysis as for spheres is performed in Sect. 6 on the surface of an oblate spheroid and a triaxial ellipsoid. This analysis was performed to answer three questions: Firstly, do these algorithms generate uniform distributions on the ellipsoid surface? Answering this requires the patch area formula derived in Sect. 2. Secondly, of those samplers found to be uniform, which one is the fastest? Thirdly, does the speed depend on the aspect ratio of the ellipsoid? For example, can the symmetry of a spheroid favour one class of sampler over another? By answering these questions, one can recommend a particular algorithm (or algorithms) to uniformly sample the ellipsoid surface.

## Surface area of an ellipsoidal patch

In polar coordinates, the ellipsoid area element is given by [21],

4 $$\begin{aligned} s(\theta , \phi ) = \sin \theta \sqrt{ b^2c^2 \sin ^2\theta \cos ^2\phi + a^2c^2 \sin ^2\theta \sin ^2\phi + a^2b^2 \cos ^2\theta } \, , \end{aligned}$$

so that infinitesimal area $\textrm{d}S$ is,

5 $$\begin{aligned} \textrm{d} S = s(\theta , \phi ) \, \textrm{d}\phi \, \textrm{d}\theta . \end{aligned}$$

Thus, the area of the ellipsoid is given by,

6 $$\begin{aligned} \begin{aligned} S&= \int \int \textrm{d}S \\&= \int _{0}^{\pi } \int _{0}^{2\pi } s(\theta , \phi ) \, \textrm{d}\phi \, \textrm{d}\theta \\&= 8\int _{0}^{\pi /2} \int _{0}^{\pi /2} s(\theta , \phi ) \, \textrm{d}\phi \, \textrm{d}\theta . \end{aligned} \end{aligned}$$

In the last equality, only the octant of the ellipsoid where $x\ge 0$, $y\ge 0$ and $z\ge 0$ is considered. This corresponds to taking $0 \le \phi \le \pi /2$ and $0 \le \theta \le \pi /2$. By the symmetry of the (general) ellipsoid, the area over the entire surface is given by the area of the octant, multiplied by 8.

The area of a patch can be obtained by replacing the limits with the values $\theta _0$, $\theta _1$, $\phi _0$ and $\phi _1$, which define the vertices of the patch. The integral of interest here is therefore,

7 $$\begin{aligned} S_{\textrm{patch}} = \int _{\theta _0}^{\theta _1} \int _{\phi _0}^{\phi _1} s(\theta , \phi ) \, \textrm{d}\phi \, \textrm{d}\theta \mathrm {.} \end{aligned}$$

### Derivation of the patch area formula

It is entirely possible to integrate the double integral in Eq. 7 numerically [25]. However, it is also possible to rewrite the double integral so that the inner integral (over $\phi$) is expressed using incomplete elliptic integrals of the second kind. Following Maas [21], one can make the transformation

8 $$\begin{aligned} \xi = \cos \theta \end{aligned}$$

to give (using that $\textrm{d}\theta =-\sin \theta \, \textrm{d}\xi$ and $\sin ^2\theta = 1 - \xi ^2$),

9 $$\begin{aligned} S_{\textrm{patch}} = \int _{\cos \theta _0}^{\cos \theta _1} \int _{\phi _0}^{\phi _1} s(\xi , \phi ) \, \textrm{d}\phi \, \textrm{d}\xi \, , \end{aligned}$$

where

10 $$\begin{aligned} s(\xi , \phi ) = -bc \left[ 1 + \xi ^2\left( \frac{a^2}{c^2} -1 \right) + \left( 1 - \xi ^2\right) \left( \frac{a^2}{c^2} -1\right) \sin ^2\phi \right] ^{1/2} . \end{aligned}$$

Note that the minus sign in Eq. 10 does not indicate a negative area. This is because $\cos \theta _1 < \cos \theta _0$, when $\theta _0 < \theta _1$ with both limits within $[0, \pi /2]$. The minus sign can thus be cancelled by interchanging the $\xi$ limits, and the resulting area is positive.

By defining

11 $$\begin{aligned} g(\xi ) = \left[ 1 + \xi ^2\left( \frac{a^2}{c^2} -1 \right) \right] ^{1/2} \, , \end{aligned}$$

and

12 $$\begin{aligned} k(\xi ) = \frac{\left[ \left( 1 - \xi ^2 \right) \left( 1 - \frac{a^2}{b^2} \right) \right] ^{1/2}}{g(\xi )} \, , \end{aligned}$$

the patch area may be expressed as,

13 $$\begin{aligned} S_\textrm{patch} = bc \int _{\cos \theta _1}^{\cos \theta _0} g(\xi ) \left[ E(\phi _1, k) - E(\phi _0, k) \right] \, \textrm{d}\xi \, , \end{aligned}$$

where

14 $$\begin{aligned} E(\psi , k) = \int _{0}^{\psi } \left( 1 - k^2 \sin ^2 \phi \right) ^{1/2} \textrm{d}\phi \end{aligned}$$

is Legendre’s incomplete elliptic integral of the second kind. If $a> b > c$, then the value of $k(\xi )$ is purely imaginary for $\xi \ne 1$. This occurs because the factor $1 - (a/b)^2$ in the numerator of Eq. 12 becomes negative when $a > b$. The elliptic integral with *k* purely imaginary can be transformed into an expression involving real valued variables [26], however this involves additional trigonometric and square root evaluations and thus requires extra computations. Instead, one can transform the problem by interchanging *a* and *b* in Eq. 13 and replacing the $\phi$ limits with $\phi _0 - \pi /2$ and $\phi _1 - \pi /2$. This rotation gives the equivalent expression for patch area,

15 $$\begin{aligned} \boxed { S_\textrm{patch} = ac \int _{\cos \theta _1}^{\cos \theta _0} \gamma (\xi ) \left[ E(\phi _1-\pi /2, \kappa ) - E(\phi _0-\pi /2, \kappa ) \right] \, \textrm{d}\xi } \, , \end{aligned}$$

where,

16 $$\begin{aligned} \gamma (\xi ) = \left[ 1 + \xi ^2\left( \frac{b^2}{c^2} -1 \right) \right] ^{1/2} \, , \end{aligned}$$

17 $$\begin{aligned} \kappa (\xi ) = \frac{\left[ \left( 1 - \xi ^2\right) \left( 1 - \frac{b^2}{a^2} \right) \right] ^{1/2} }{\gamma (\xi )} \, . \end{aligned}$$

When $a > b$, Eq. 15 should be used for patch area, while Eq. 13 gives the appropriate formula for the $b > a$ case. As for the choice of *c*, note that the value of $g(\xi )$ is real for any positive values of *a* (or *b*) and *c*, as the second term in Eq. 11 is always greater than $-1$ as $\xi ^2 \in [0,1]$. Therefore, in Eqs. 13 and 15, *c* can take any positive value (whether smaller or greater than *a* or *b*).

The elliptic integral $E(\psi , k)$ can be efficiently evaluated by writing it in terms of Carlson’s elliptic integrals,

18 $$\begin{aligned} E(\psi , k) = \sin \psi \, R_F(u,v,w) - \frac{1}{3}k^2\sin ^3\psi R_D(u,v,w) \, , \end{aligned}$$

with

19 $$\begin{aligned} u&= \cos ^2\psi \, , \end{aligned}$$

20 $$\begin{aligned} v&= 1-k^2\sin ^2\psi \, ,\end{aligned}$$

21 $$\begin{aligned} w&= 1 \, , \end{aligned}$$

where $R_F$ and $R_D$ are the Carlson elliptic integrals of the first and second kind, respectively. These integrals can be efficiently computed numerically, using algorithms based on duplication theorems [27, 28]. If computational speed is a priority, then one could alternatively compute $E(\psi , k)$ using the faster method developed by Fukushima [29, 30].

Given an efficient algorithm to compute $E(\psi , k)$, evaluation of Eq. 15 can be interpreted as a one-dimensional integration problem. This can be readily solved to the required accuracy using an algorithm such as Gaussian quadrature or Romberg integration [25, 31], the latter of which was used in this study.

The patch area formula given in Eq. 15 assumes that both the $\theta$ and $\phi$ limits are within the interval $[0, \pi /2]$. Outside of this interval, it is possible to obtain unexpected negative values of patch area (for example, when using Eq. 18 for $\phi > \pi$) that lead to unwanted cancellations. However, the $[0, \pi /2]$ interval is sufficient to calculate any patch area on the ellipsoid by exploiting its eight-fold symmetry. By this symmetry, the area of a patch entirely contained within the $x, y, z \ge 0$ octant of the surface equals the area for reflected versions of the patch in the other octants. If the set of patches have been chosen in such a way that a limit occurs halfway through a patch, then that patch area can be determined by calculating the area of the half-patch within the limits and doubling the answer. For patches containing both a $\theta$ and a $\phi$ limit, one need only calculate the quarter of the patch area within the limits and multiply by 4. These cases together account for all patches on the surface, provided that the number of distinct $\phi$ values is chosen to be even, so that the set of patches match the symmetry of the ellipsoid (the number of $\theta$ values can be odd or even). By exploiting the symmetry of the ellipsoid in this way, many redundant computations are avoided. This can be useful if one wishes to obtain accurate areas by setting a low tolerance value in the chosen numerical integration algorithm.

### Spheres and spheroids

For a sphere of radius *a*, the patch area integral simplifies to,

22 $$\begin{aligned} S_{\textrm{patch}}&= \int _{\theta _0}^{\theta _1} \int _{\phi _0}^{\phi _1} a^2 \sin \theta \, \textrm{d}\phi \, \textrm{d}\theta \mathrm {.} \end{aligned}$$

23 $$\begin{aligned}&= a^2 (\cos \theta _0 - \cos \theta _1) (\phi _1 - \phi _0) \, . \end{aligned}$$

For a spheroid with $a = b \ne c$, an analytical expression for patch area can be derived. Starting from the triaxial patch area given in Eq. 15, it can immediately be seen from Eq. 17 that $\kappa (\xi )$ vanishes when $a= b$. For $\kappa = 0$, the elliptic integral $E(\psi , \kappa )$ reduces to,

24 $$\begin{aligned} E(\psi , \kappa ) = \int _{0}^{\psi } (1 - 0)^{1/2} \textrm{d} \phi = \psi \, . \end{aligned}$$

Thus,

25 $$\begin{aligned} S_{\textrm{patch}} = ac(\phi _1 - \phi _0) \int _{\cos \theta _1}^{\cos \theta _0} \left( 1 + q^2\xi ^2 \right) ^{1/2} \textrm{d} \xi \, , \end{aligned}$$

where,

26 $$\begin{aligned} q^2 = \frac{a^2}{c^2} - 1 \, . \end{aligned}$$

To solve this integral, one can make the substitution $\sinh u = q \xi$ to obtain,

27 $$\begin{aligned} S_{\textrm{patch}} = \frac{ac(\phi _1 - \phi _0)}{q} \int _{u_0}^{u_1} \cosh ^2 u \, \textrm{d}u \, , \end{aligned}$$

with $u_0 = \textrm{arcsinh} (q\cos \theta _1)$ and $u_1 = \textrm{arcsinh} (q\cos \theta _0)$. Using that,

28 $$\begin{aligned} \int \cosh ^2 x \, \textrm{d} x = \int \frac{1+\cosh 2x}{2} \textrm{d} x = \frac{x}{2} + \frac{\sinh (2x)}{4} + C \, , \end{aligned}$$

and

29 $$\begin{aligned} \sinh 2u = 2 \sinh u \cosh u = 2 q\xi \sqrt{1 + q^2\xi ^2} \, , \end{aligned}$$

the spheroid patch area is given by,

30 $$\begin{aligned} \begin{aligned} S_{\textrm{patch, oblate}}&= \frac{ac(\phi _1 - \phi _0)}{2q} \Bigl [ \textrm{arcsinh}(q\cos \theta _0) - \textrm{arcsinh}(q\cos \theta _1) \\&\quad + q\cos \theta _0\sqrt{1 + q^2 \cos ^2\theta _0} - q\cos \theta _1\sqrt{1 + q^2 \cos ^2\theta _1} \Bigr ] \, . \end{aligned} \end{aligned}$$

Equation 30 is named as ‘oblate’ because the quantity *q* is only real valued when $a > c$. In the prolate case, *q* is imaginary valued. Using the relation $\sinh (ix) = i\sin x$, the patch area of a prolate spheroid can be written as,

31 $$\begin{aligned} \begin{aligned} S_{\textrm{patch, prolate}}&= \frac{ac(\phi _1 - \phi _0)}{2\bar{q}} \Bigl [ \textrm{arcsin}(\bar{q}\cos \theta _0) - \textrm{arcsin}(\bar{q}\cos \theta _1) \\&\quad + \bar{q}\cos \theta _0\sqrt{1 + \bar{q}^2 \cos ^2\theta _0} - \bar{q}\cos \theta _1\sqrt{1 + \bar{q}^2 \cos ^2\theta _1} \Bigr ] \, , \end{aligned} \end{aligned}$$

where $$\bar{q}^2 = 1 - a^2/c^2$$ (i.e. $$\bar{q} = iq$$).

## Speed and uniformity of the sampling algorithms

Ellipsoid patch areas calculated using Eqs. 15 (for ellipsoids) and 23 (for spheres) were used to investigate uniformity (with respect to surface area) of the sampling algorithms to be discussed in Sects. 4 and 5. For each algorithm studied, a sample of $N = 10^8$ random points was generated using implementations written in C++, run using the Linux Subsystem for Windows and analysed using a Python script. This was done for two different random number generators (RNG):

- Lagged Fibonacci (with 4 feedback shifts and the exclusive-or operation).
- YARN5 generator.

The implementations used in this work are from the TRNG library^1^ (using the lagfib4xor_19937_64 and yarn5 classes, respectively) [32]. The following analysis was done with two different RNG algorithms to verify that the source of random numbers itself has no influence on the final results. This is done by checking that the results from each generator are similar.

To study each sampling algorithm, the following quantities were measured:

- Run-time, $$t_\textrm{run}$$.

For each algorithm (and choice of RNG), the required run-time was measured using the ctime header from the C++ Standard Library.

- Relative speed.

Since the raw run-time depends on many factors; such as language, operating system and CPU, the relative speed of each algorithm was computed from the measured run-times. The speed relative to a given benchmark algorithm may be computed as $t_\textrm{benchmark} / t$, where $t_\textrm{benchmark}$ is the run-time of the benchmark and *t* is the run-time for the desired algorithm.

- Acceptance rate, *r*.

For those algorithms that use rejection sampling, the acceptance rate is the proportion of all trial points that were accepted. In cases where rejection sampling is not employed, one obtains a value of $r=1$, i.e. all generated points were accepted.

The above quantities can be used to evaluate the speed of each sampling method. These were measured by a program that generated the $10^8$ points and nothing else. Thus, the measured run-times in this Paper constitute only the time required to generate the points.

To investigate the uniformity of these algorithms, another set of $N=10^8$ points was generated for each method. In this case, the points were binned into one of a set of patches (as defined by Fig. 1). This was done by defining patches with increments of one degree in both angular coordinates $\theta$ and $\phi$. This gives 181 distinct values of $\theta$ and 360 values of $\phi$. Since two of the $\theta$ values correspond to caps at the poles, there are a total of

32 $$\begin{aligned} n = (181-2)\times 360 + 2 = 64\,442 \end{aligned}$$

patches (i.e. $64\,442$ bins) defined on the surface. Given a point in polar coordinates, the relevant $\theta$ and $\phi$ indices can be found by comparing each coordinate to a list of the values defining each bin. For the sake of speed, these comparisons were here performed using Bottenbruch’s version of the binary search algorithm [33, 34].

- Relative standard deviation, RSD.

Given a binned distribution of random surface points, one can calculate number of points per area for each patch. From this distribution, a mean and a standard deviation can be computed. The relative standard deviation is then just the standard deviation divided by the mean. This quantity is interpreted here as a measure of non-uniformity, with a large value indicating high deviation from the mean. Since these distributions are expected to be uniform after dividing by patch areas, one can expect the relative standard deviation to be small.

- Chi-squared, $$\chi ^2$$.

Another way to measure uniformity (or lack thereof) is to perform a $\chi ^2$ test for goodness of fit [25, 35]. This uses the $\chi ^2$ statistic, defined as

33 $$\begin{aligned} \chi ^2 = \sum _{i}^{n} \frac{ \left( O_i - E_i\right) ^2 }{ E_i } \, , \end{aligned}$$

where index *i* refers to a particular patch, $O_i$ is the observed number of random points within patch *i* and $E_i$ is the expected number of points within patch *i*. Knowing the area of each patch, one can calculate $E_i$; given the null hypothesis that this number divided by surface area is uniform. This is given by,

34 $$\begin{aligned} E_i = \frac{ N S_i }{ \sum _{i}^{n} S_i } \, , \end{aligned}$$

where $S_i$ is the surface area of patch *i* and $\sum _{i}^{n} S_i$ is the sum of all patch areas. Given $\chi ^2$, the validity of the null hypothesis can be tested by comparing this value to a critical value, $\chi _\textrm{crit}^2$, which is determined using a chi-squared distribution function, with *n* degrees of freedom. For $n = 64\,442$ degrees of freedom (i.e. equal to the number of bins) the critical value at the 5% confidence level is found to be (using the chi2.ppf function from the Python stats module).

35 $$\begin{aligned} \chi _\textrm{crit}^2 = 65\,033.6 \, . \end{aligned}$$

If a measured $\chi ^2$ statistic exceeds the critical value, then there is sufficient evidence to reject the null hypothesis and conclude that the distribution is non-uniform. On the other hand, a $\chi ^2$ value smaller than critical cannot prove the null hypothesis, but does provide some confidence that the sample could be uniform.

## Uniform sampling on the surface of a sphere

Before proceeding to investigate ellipsoid surface samplers, it is necessary to have a reliable method for generating uniform random points on the surface of a sphere. A simple approach, based on a two-dimensional circle rejection sampling method given by von Neumann [36], is to generate points in a unit length cube. Points that have magnitude smaller than unity (i.e. that lie within a unit sphere) are accepted and scaled to lie on the required sphere surface.

While this approach suffices, other algorithms exist that are faster. In this work, six sphere sampling algorithms (including the cubic rejection method) were considered. The other five are: Marsaglia’s improved method [12], the ‘trig method’ (so-called due to use of trigonometric functions) [8, 15], use of Gaussian random numbers [12, 13] (generated here using Doornik’s implementation of the ziggurat method [37, 38]), Cook’s method [12, 14] and rejection sampling using the surface area element at a point [3]. For details on how these algorithms work, see the given References. The surface area rejection sampling method used here is a special case of an algorithm for ellipsoids, described in more detail in Sect. 5.

### Comparison of the sphere sampling algorithms

The quantities described in Sect. 3 were measured for samples of $N=10^8$ randomly generated points on the unit sphere. The results of this are shown in Table 1. Considering first the speed (relative to the cubic rejection method) of each algorithm, it is clear that the fastest is that of Marsaglia, which has the shortest run-time and highest relative speed, regardless of random number generator. This is despite the fact that both the trig and Gaussian methods have a perfect acceptance rate. The speed of Marsaglia’s method lies in the fact that no costly trigonometric or Gaussian deviate calculations are required.

**Table 1:** Results of using various sphere surface samplers to generate $10^8$ points, using two different random number generators. Each column shows (from left to right): algorithm name, run-time (in seconds), speed relative to cubic rejection, acceptance rate, relative standard deviation (as a percentage), the $\chi ^2$ statistic and whether $\chi ^2$ is smaller than the critical value, $\chi _\textrm{crit}^2 = 65\,033.6$

**Sphere**: $(a,b,c) = (1,1,1)$						
RNG	**Lagged Fibonacci** (lagfib4xor)					
**Algorithm**	$t_\textrm{run}$ (s)	speed	*r*	RSD (%)	$\chi ^2$	$\chi ^2 < \chi _\textrm{crit}^2$
Cubic Rej	2.803	1.0	0.52364	3.741	63834.1	Yes
Marsaglia	1.205	2.326	0.78545	3.783	64958.6	Yes
Trig	2.345	1.195	1.0	3.735	64346.7	Yes
Gaussian	3.477	0.806	1.0	3.736	64059.1	Yes
Cook	4.493	0.624	0.30844	3.725	64454.0	Yes
Area Rej	5.513	0.508	0.63656	3.729	64260.0	Yes

RNG	**YARN5s** (yarn5s)					
**Algorithm**	$t_\textrm{run}$ (s)	speed	*r*	RSD (%)	$\chi ^2$	$\chi ^2 < \chi _\textrm{crit}^2$
Cubic Rej	7.226	1.0	0.52356	3.738	63920.5	Yes
Marsaglia	2.999	2.409	0.78543	3.726	64931.0	Yes
Trig	4.018	1.798	1.0	3.737	64792.5	Yes
Gaussian	7.367	0.981	1.0	3.723	64339.9	Yes
Cook	14.008	0.516	0.3084	3.769	64724.5	Yes
Area Rej	8.027	0.9	0.63664	3.704	64468.3	Yes

It is also of note that the relative speed of each algorithm differs based on the choice of random number generator. For lagged Fibonacci, which is faster than YARN5, Marsaglia’s method is nearly twice as fast as the trig method. With the YARN5 generator, Marsaglia’s method remains fastest, but the gap compared to the trig method is smaller. It is thus conceivable that for a much slower source of random numbers, the trig method could be advantageous.

**Fig. 2:**
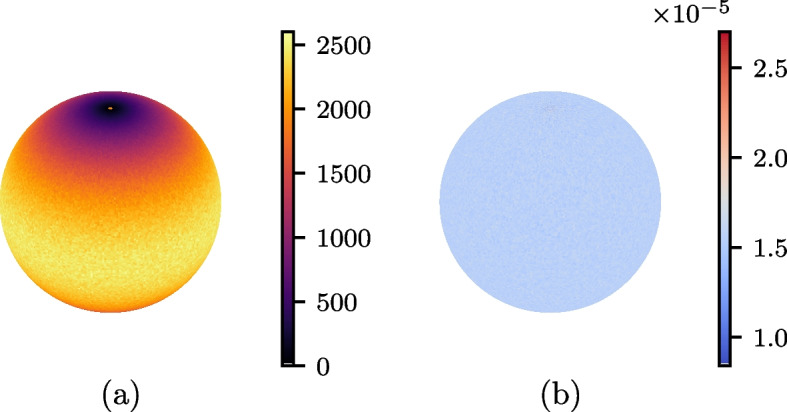
Distribution of random points on a unit sphere, generated using Marsaglia’s method with random numbers obtained by the lagged Fibonacci algorithm. **a** Number of points per patch. **b** Selection probability per patch area, with patch areas computed using Eq. 23

As far as uniformity is concerned, every sample yields a relative standard deviation of roughly $3.7\%$, and a $\chi ^2$ value just under the critical value (see Eq. 35). This may suggest that the distributions are indeed uniform. To shed further light on the uniformity (or non-uniformity) of the obtained samples, one could visually inspect the distributions. Figure 2 shows the distributions obtained using Marsaglia’s method with the lagged Fibonacci generator. The raw number of points per patch is greater at the equator than near the poles (with a high value at the patches containing the poles themselves). The patches with more hits are those with greater surface area, as is verified when looking at number of points per patch area. Now a much more uniform distribution presents itself.

The use of relative standard deviations and $\chi ^2$ tests allow uniformity to be assessed without the need to produce plots for each sample. The corresponding values in Table 1 do not give evidence for non-uniformity, with small standard deviations relative to the mean and all $\chi ^2$ tests passing. This is expected since the algorithms are all designed to produce uniformly random points.

## Uniform sampling on the surface of an ellipsoid

**Fig. 3:**
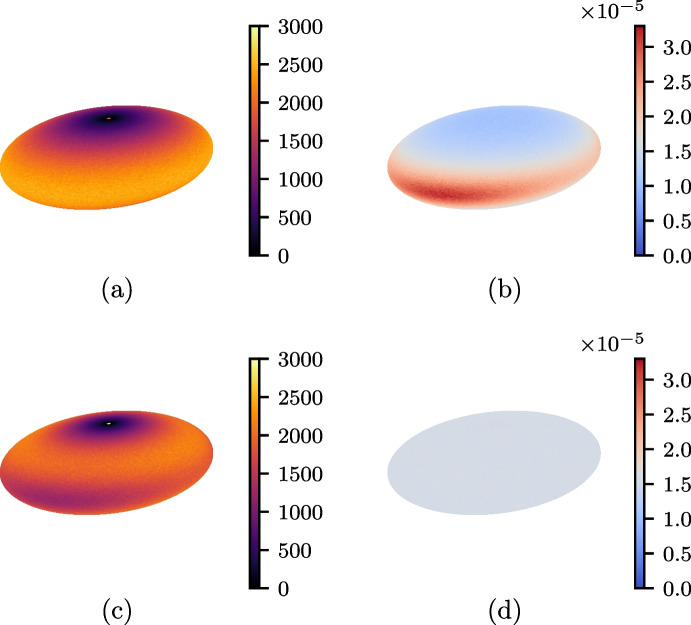
Distribution of random points on a triaxial ellipsoid, with random numbers obtained by the lagged Fibonacci algorithm. **a** and **b**: Number of points per patch and selection probability per patch area respectively, using the naive scaling method (with Marsaglia’s sphere sampler). **c** and **d**: Number of points per patch and selection probability per patch area respectively, using the gradient rejection method. Patch areas were computed using Eq. 15

Suppose one has a point on a unit sphere that was generated using a uniform sampling algorithm, such as one of those discussed in Sect. 4. This point can easily be transformed so it lies on the surface of an ellipsoid of axis lengths *a*, *b* and *c* by scaling its Cartesian coordinates by each axis respectively. This ‘naive method’ is simple and only requires a sphere sampler. However, it does not result in a uniform distribution of points on the ellipsoid surface. Instead, there is a greater concentration of points near the poles of the major and middle axes as shown by Figs. 3(a) and (b). By using a uniform ellipsoid surface sampler, a sample such as that of Figs. 3(c) and (d) can be obtained.

In this section, three different rejection sampling algorithms for generating uniform distributions of points on the ellipsoid surface are explained. These are then studied in Sect. 6, using the measures discussed in Sect. 3.

### Gradient vector rejection sampling

One approach to generate uniform samples on the ellipsoid surface is to first generate points on the unit sphere, perform anisotropic scaling to obtain trial points, $\textbf{p}(x,y,z)$, on the ellipsoid and then reject some of them in such a way that the accepted points are uniformly distributed. Chen and Glotzer give such a method for prolate spheroids, based on the work of Williamson, using the magnitude of the gradient vector of the ellipsoid surface [1, 8]. This approach is easily generalised to the case of any ellipsoid, as is demonstrated here.

The rejection sampling works by defining a function giving the probability of accepting a trial point,

36 $$\begin{aligned} P(\textrm{accept}) \equiv \frac{g(x,y,z)}{g_\textrm{max}} \, , \end{aligned}$$

where the function *g*(*x*, *y*, *z*) is given by,

37 $$\begin{aligned} \frac{g(x,y,z)}{g_\textrm{max}} = \frac{\left| \textbf{n} \right| }{\left| \textbf{n} \right| _\textrm{max}} = \frac{\left| \nabla f \right| }{\left| \nabla f \right| _\textrm{max}} \, . \end{aligned}$$

The vector $\textbf{n}$ is the normal to the surface at point (*x*, *y*, *z*), and $\nabla f$ is the gradient vector. The function *f* defines the surface of the ellipsoid,

38 $$\begin{aligned} f(x,y,z) = \frac{x^2}{a^2} + \frac{y^2}{b^2} + \frac{z^2}{c^2} \, , \end{aligned}$$

with the surface being the set of points where $f(x,y,z) = 1$. The magnitude of the gradient vector is given by,

39 $$\begin{aligned} \left| \nabla f \right| = 2\sqrt{\frac{x^2}{a^4} + \frac{y^2}{b^4} + \frac{z^2}{c^4}} \, . \end{aligned}$$

Since the acceptance probability is the ratio of this expression to its maximum value, the prefactor of 2 may be ignored. With this factor dropped, the expression can be used as function *g*(*x*, *y*, *z*).

To use this expression, the maximum of the function

40 $$\begin{aligned} \begin{aligned} g&= \sqrt{\frac{x^2}{a^4} + \frac{y^2}{b^4} + \frac{z^2}{c^4}} \,, \\&= \sqrt{\frac{\sin ^2\theta \cos ^2\phi }{a^2} + \frac{\sin ^2\theta \sin ^2\phi }{b^2} + \frac{\cos ^2\theta }{c^2}} \,, \end{aligned} \end{aligned}$$

must be found. The latter equality in Eq. 40 arises due to conversion of coordinates from Cartesian to scaled spherical polars, using Eqs. 1-3. Use of polar coordinates gives a more natural parametrisation of the function to be maximised. The partial derivatives of $g(\theta ,\phi )$ can be determined using the chain rule,

41 $$\begin{aligned} \frac{\partial g}{\partial \theta }&= \frac{1}{g} \sin \theta \cos \theta \left( \frac{\cos ^2\phi }{a^2} + \frac{\sin ^2\phi }{b^2} - \frac{1}{c^2} \right) \, , \end{aligned}$$

42 $$\begin{aligned} \frac{\partial g}{\partial \phi }&= \frac{1}{g} \sin \phi \cos \phi \sin ^2\theta \left( \frac{1}{b^2} - \frac{1}{a^2} \right) \, . \end{aligned}$$

At the extreme points of $g(\theta ,\phi )$, both partial derivatives equal zero. To make the right-hand side of Eq. 41 vanish, either $\sin \theta$, $\cos \theta$ or the term in brackets must be zero. This is equivalent to the condition,

$$\begin{aligned} \theta = 0 \mathrm {, \,{\textbf {or}}\,, } \, \theta = \pi /2 \mathrm {, \,{\textbf {or}}\,, } \, \theta = \pi \mathrm {, \,{\textbf {or}}\,, } \, \sin ^2\phi \left( \frac{1}{b^2} - \frac{1}{a^2}\right) = \frac{1}{c^2} - \frac{1}{a^2} \, . \end{aligned}$$

The first three cases simply state that the point is either a *c*-axis pole or an equator point. The last case is satisfied for any point on a sphere. For Eq. 42, one requires either $\sin \phi =0$, $\cos \phi =0$, $\sin \theta =0$ or $a=b$, i.e.

$$\begin{aligned} \phi = 0 \mathrm {, \,{\textbf {or}}\,, } \, \phi = \pi \mathrm {, \,{\textbf {or}}\,, } \, \phi = \pi /2 \mathrm {, \,{\textbf {or}}\,, } \, \phi = 3\pi /2 \mathrm {, \,{\textbf {or}}\,, } \, \theta = 0 \mathrm {, \,{\textbf {or}}\,, } \, \theta = \pi \mathrm {, \,{\textbf {or}}\,, } \, a=b \, . \end{aligned}$$

The latter case says that the $\phi$ derivative vanishes at any point on a spheroid, where *c* is the distinct axis. The remaining cases require particular values of $\phi$ or $\theta$. In order to make both partial derivatives vanish simultaneously on an ellipsoid with arbitrary axes, there are only six possible points. These points are the poles of the *a*, *b* and *c* axes. Therefore, the maximum value of *g* is given by,

43 $$\begin{aligned} g_\textrm{max} = \frac{1}{\min (a,b,c)} \, . \end{aligned}$$

Substituting Eqs. 40 and 43 into Eq. 36 gives the following expression for the acceptance probability of a trial point,

44 $$\begin{aligned} P(\textrm{accept}) = \min (a,b,c) \sqrt{\frac{x^2}{a^4} + \frac{y^2}{b^4} + \frac{z^2}{c^4}} \, . \end{aligned}$$

Equation 44 forms the basis of a rejection sampling algorithm using the gradient vector at a trial point generated by the ‘naive method’. This procedure is summarised via pseudocode in Algorithm 1.

**Algorithm 1:**
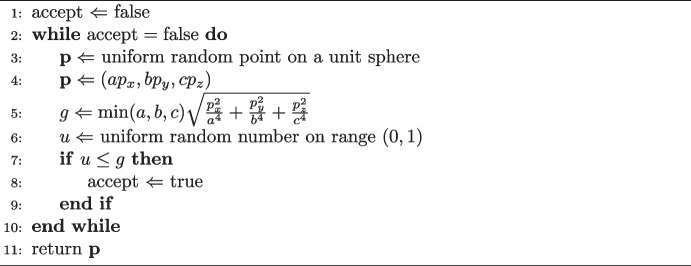
The gradient rejection algorithm on an ellipsoid with axis lengths *a*, *b* and *c*.

### Area element rejection sampling

A natural approach to generating random points that are uniform with respect to surface area is to make use of the expression for the surface area element, *s*. Following Melfi and Schoier [3], suppose an area element is given in terms of arbitrary coordinates $(\mu , \nu )$. Values of $\mu$ and $\nu$ are obtained using a uniform random number generator. One can then define an acceptance probability,

45 $$\begin{aligned} P(\textrm{accept}) = \frac{s(\mu ,\nu )}{s_\textrm{max}} \, , \end{aligned}$$

where $s_\textrm{max}$ is the maximum value of the surface area element.

One could try selecting Cartesian coordinates *x* and *y* and use points (*x*, *y*, *f*(*x*, *y*)), where

46 $$\begin{aligned} f(x,y) = c\sqrt{1 - x^2/a^2 - y^2/b^2)} \, . \end{aligned}$$

The surface area element is then given by,

47 $$\begin{aligned} s(x,y) = \sqrt{1 + \left( \frac{\partial f}{\partial x} \right) ^2 + \left( \frac{\partial f}{\partial y} \right) ^2 } \, . \end{aligned}$$

The partial derivatives,

48 $$\begin{aligned} \frac{\partial f}{\partial x}&= \frac{-c^2 x}{a^2 f(x,y)} \, ,\end{aligned}$$

49 $$\begin{aligned} \frac{\partial f}{\partial y}&= \frac{-c^2 y}{b^2 f(x,y)} \, , \end{aligned}$$

are not defined at values where $z = f(x,y)$ vanish (i.e. the equator, $\theta =\pi /2$), due to division by zero. Therefore, Cartesian coordinates are unsuitable for this algorithm.

Instead, polar coordinates $(\theta , \phi )$ can be used. The surface area element is given by Eq. 4, and so the acceptance probability is

50 $$\begin{aligned} P(\textrm{accept}) = \frac{ \sin \theta \sqrt{b^2c^2 \sin ^2\theta \cos ^2\phi + a^2c^2 \sin ^2\theta \sin ^2\phi + a^2b^2 \cos ^2\theta } }{s_\textrm{max}} \, . \end{aligned}$$

The value of the maximum, derived in Appendix 1, depends on the choice of semi-axis lengths. Defining,

51 $$\begin{aligned} \beta = \frac{b^2}{2(b^2-c^2)} \, , \end{aligned}$$

the maximum is given by,

52 $$\begin{aligned} s_\textrm{max} = {\left\{ \begin{array}{ll} \beta \left( a^2c^2\beta - a^2b^2\beta + a^2b^2 \right) , &{} \text {if triaxial or oblate,} \\ ac, &{} \text {otherwise.} \\ \end{array}\right. } \end{aligned}$$

Equations 50 and 52 can be used as the basis of an area rejection sampling method, as shown in Algorithm 2. Values of $\theta$ and $\phi$ are randomly chosen using uniform generators and the area element used to determine whether to accept the corresponding point.

**Algorithm 2:**
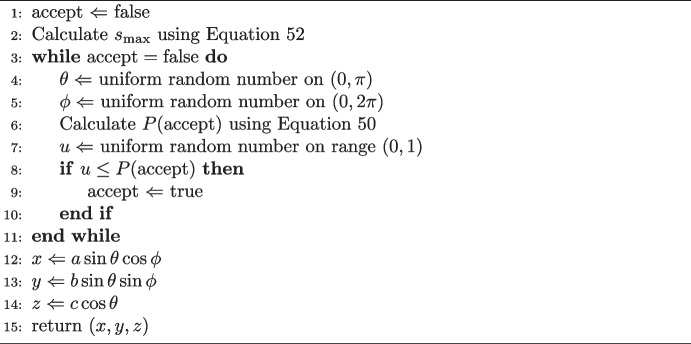
The polar coordinate area rejection algorithm on an ellipsoid with axis lengths *a*, *b* and *c*.

When dealing with a sphere or a spheroid, the area rejection algorithm becomes computationally simpler. This is because the area element becomes,

53 $$\begin{aligned} s_\textrm{spheroid}(\theta ,\phi ) = \sin \theta \sqrt{a^2 c^2 \sin ^2\theta + a^4 \cos ^2\theta } \, , \end{aligned}$$

for a spheroid and

54 $$\begin{aligned} s_\textrm{sphere}(\theta ,\phi ) = a^2 \sin \theta \, , \end{aligned}$$

for a sphere. In both cases, there is no dependence on $\phi$. This means that the random generation of $\phi$ can be taken outside the loop in Algorithm 2. Combined with a simpler form of the area element, this means fewer computations are required.

Another area rejection algorithm can be created by selecting another coordinate system. In this work, use of the Mercator parametrisation (*u*, *v*), where,

55 $$\begin{aligned} x&= a\,\textrm{sech}\, v \cos u \, , \end{aligned}$$

56 $$\begin{aligned} y&= b\,\textrm{sech}\, v \sin u \, , \end{aligned}$$

57 $$\begin{aligned} z&= c\tanh u \, , \end{aligned}$$

was studied. The *u* coordinate is defined similarly to the polar $\phi$ coordinate and exists on the range $[0,2\pi ]$. However, the *v* coordinate is defined on range $(-\infty , \infty )$. By truncating to a finite range, it is possible to obtain an approximately uniform sampling algorithm using these coordinates. Here, the finite range $(-2\pi ,2\pi )$ was used. Using the coefficients of the first fundamental form, the area element can be found to be,

58 $$\begin{aligned} s(u,v) = \,\textrm{sech}^2\, v \sqrt{a^2 b^2 (1 - \,\textrm{sech}^2\,v) + c^2 \,\textrm{sech}^2\,v\,(a^2 \sin ^2 u + b^2 \cos ^2 u)} \, . \end{aligned}$$

Since this expression requires only one hyperbolic and one trigonometric function evaluation to compute (as $\cos ^2 u = 1 - \sin ^2 u$), a single evaluation of *s* could be expected to be faster here than for polar coordinates. The partial derivatives are,

59 $$\begin{aligned} \frac{\partial s}{\partial u}&= \frac{c^2 \textrm{sech}^4 v \sin u \cos u (a^2-b^2)}{w(u,v)} \, , \end{aligned}$$

60 $$\begin{aligned} \frac{\partial s}{\partial v}&= 2 \,\textrm{sech}^2\, v \tanh v \left( \frac{\textrm{sech}^2\, v}{2w(u,v)} (a^2 b^2 - c^2(a^2 \sin ^2 u + b^2 \cos ^2 u)) - w(u,v) \right) \, , \end{aligned}$$

where,

61 $$\begin{aligned} w(u,v) = \sqrt{a^2 b^2 (1 - \,\textrm{sech}^2\,v) + c^2 \,\textrm{sech}^2\,v\,(a^2 \sin ^2 u + b^2 \cos ^2 u)} \, . \end{aligned}$$

The *v* derivative vanishes when $\textrm{sech}\, v = 0$ or $\tanh v = 0$ (The bracketed term is always positive for $a>b>c$). The former case requires infinite *v*, in which limit the area element vanishes. The latter case is equivalent to $v = 0$, which corresponds to the equator (i.e. $z=0$). Given that $v=0$, the only way to make the *u* derivative equal zero is to make *u* a multiple of $2\pi$ (so $\sin u = 0$ or $\cos u = 0$). The maximum possible value of *s* is thus obtained by taking $u = \pi /2$ or $3\pi /2$, so that $\sin ^2 u = 1$ and therefore,

62 $$\begin{aligned} s_\textrm{max} = ac \, . \end{aligned}$$

The acceptance probability in these coordinates is given by,

63 $$\begin{aligned} P(\textrm{accept}) = \frac{\textrm{sech}^2\, v \times w(u,v)}{ac} \, . \end{aligned}$$

The process for generating random points is similar to that for polar coordinates, as shown by Algorithm 3.

**Algorithm 3:**
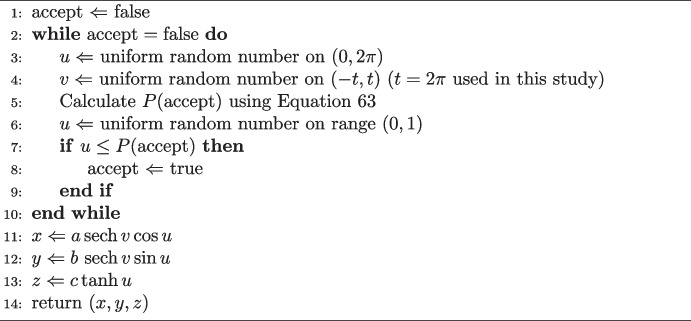
The Mercator coordinate area rejection algorithm on an ellipsoid with axis lengths *a*, *b* and *c*.

### Ray intersection sampling

The final method considered here is the generic surface sampler of Detwiler et al. [4]. An equivalent formulation of this algorithm also appears in the work of Palais et al. [6]. The method works by starting with a randomly generated point on the bounding sphere for the surface of interest. For an ellipsoid, this sphere has radius $r = \max (a,b,c)$. A uniform random point, $\mathbf {\rho }_0$ is then generated in a disk of radius *r*. Intersections are then sought between the surface and a ray orthogonal to the disk, starting at $\mathbf {\rho }_0$. If any intersections are found, one of them is randomly selected and returned as the generated surface point. If no intersections exist, the process is re-attempted until a valid intersection is found.

The method is summarised as pseudocode in Algorithm 4. Firstly, the random sphere point, $\textbf{n}$, is generated. This vector is normal to the sphere surface. Next, the orientation of the tangent disk is represented through two mutually orthogonal vectors in the disk. One of these, $\textbf{u}$, is constructed as orthogonal to $\textbf{n}$; while the other, $\textbf{v}$ can be made orthogonal to both $\textbf{n}$ and $\textbf{u}$ by taking a cross product. For subsequent calculations, $\textbf{u}$ and $\textbf{v}$ must be normalised.

A point in the disk must then be generated. This requires a random radius *B* and a random angle $\psi$ (between 0 and $2\pi$). To ensure uniformly random points in the disk, one must take the square root of a uniform random number on range (0,1) and then multiply by the desired radius [39]. Given point $(b_0, b_1)$ in two-dimensions, the 3D point $\mathbf {\rho }$ in the disk is given by,

64 $$\begin{aligned} \mathbf {\rho } = r\textbf{n} + b_0\textbf{u} + b_1\textbf{v} \, . \end{aligned}$$

**Algorithm 4:**
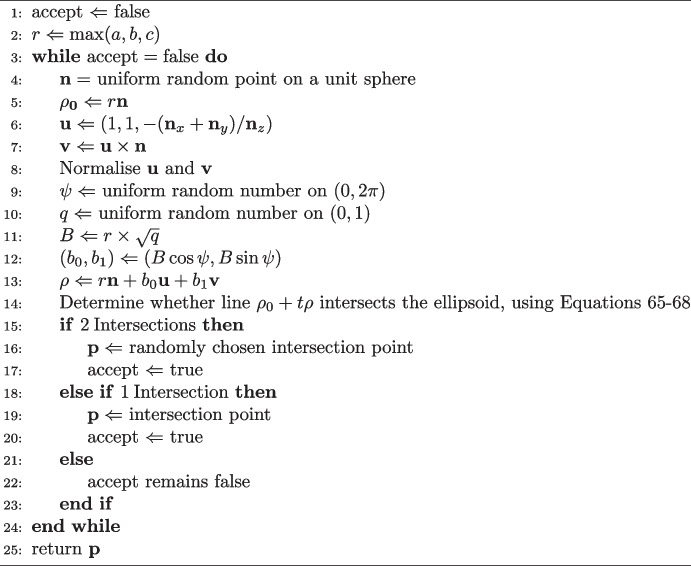
The ray intersection algorithm on an ellipsoid with axis lengths *a*, *b* and *c*.

Given point $\mathbf {\rho }$, the line $\mathbf {\rho _0} + t\mathbf {\rho }$ can be defined, where $\mathbf {\rho _0} = r\textbf{n}$ and *t* is a real number. With the ellipsoid oriented with semi-axes *a*, *b*, *c* along the *x*, *y*, *z* directions respectively, the problem of determining whether the line intersects the ellipsoid can be represented as a quadratic equation in parameter *t*, so that

65 $$\begin{aligned} t = \left( -\beta \pm \sqrt{\beta ^2 - 4\alpha \gamma } \right) / 2\alpha \end{aligned}$$

where,

66 $$\begin{aligned} \alpha&= \textbf{n}_x^2 / a^2 + \textbf{n}_y^2 / b^2 +\textbf{n}_z^2 / c^2 \, , \end{aligned}$$

67 $$\begin{aligned} \beta&= -2\rho _{0,x}\textbf{n}_x / a^2 - 2\rho _{0,y}\textbf{n}_y / b^2 - 2 \rho _{0,z}\textbf{n}_z / c^2 \, ,\end{aligned}$$

68 $$\begin{aligned} \gamma&= \rho _{0,x}^2 / a^2 + \rho _{0,y}^2 / b^2 + \rho _{0,z}^2 / c^2 \, . \end{aligned}$$

There are three possible outcomes based on the sign of the discriminant: two intersections; one intersection; no intersection. In the latter case, the calculation is repeated with new random numbers until an intersection is found. If two intersection points are obtained, a random number can be drawn to decide which one to return.

## Comparison of the ellipsoid sampling algorithms

Each of the aforementioned algorithms were used to generate a sample of $N=10^8$ random points, and were analysed as discussed in Sect. 3. Eight separate algorithms were used, including different variations of those outlined above. Along with the ‘naive scaling method’ and the ray intersection method, three variations of the gradient rejection algorithm were studied,

- Using Marsaglia’s method to generate sphere points.
- Using the ‘trig method’ to generate sphere points.
- Converting to polar coordinates prior to output (using Marsaglia’s method for spheres).

Three variations of the area rejection method were also used,

- Using polar coordinates, converting the output to Cartesians.
- Using polars, without converting to Cartesians.
- Using Mercator coordinates (with conversion to Cartesians).

The decision to consider implementations of the gradient and area rejection methods with Cartesian and polar outputs was made to study the effect this has on run-time. If one is interested in generating polar coordinates for uniform random surface points, is it faster to use an algorithm that directly yields polar coordinates?

**Table 2:** Results of using various ellipsoid surface samplers to generate $10^8$ points on an oblate spheroid, using two different random number generators. Each column shows (from left to right): algorithm name, run-time (in seconds), speed relative to naive rejection, acceptance rate, relative standard deviation (as a percentage of the mean), the $\chi ^2$ statistic and whether $\chi ^2$ is smaller than the critical value, $\chi _\textrm{crit}^2 = 65\,033.6$

**Oblate Spheroid**: $(a,b,c) = (3,3,1.5)$						
RNG	**Lagged Fibonacci** (lagfib4xor)					
**Algorithm**	$t_\textrm{run}$ (s)	speed	*r*	RSD (%)	$\chi ^2$	$\chi ^2 < \chi _\textrm{crit}^2$
Naive Scale	1.493	1.0	1.0	24.878	5005750.1	No
Grad Rej	4.284	0.349	0.69007	3.316	64726.9	Yes
Grad (Trig)	5.83	0.256	0.69009	3.336	64634.3	Yes
Grad (Pol)	9.447	0.158	0.69014	3.318	64978.8	Yes
Area Rej	4.995	0.299	0.76086	3.307	64426.4	Yes
Area (Pol)	3.223	0.463	0.76099	3.303	64032.3	Yes
Area (Merc)	10.794	0.138	0.21841	3.461	73037.1	No
Ray Method	9.281	0.161	0.69011	3.309	64192.8	Yes

RNG	**YARN5s** (yarn5s)					
**Algorithm**	$t_\textrm{run}$ (s)	speed	*r*	RSD (%)	$\chi ^2$	$\chi ^2 < \chi _\textrm{crit}^2$
Naive Scale	3.155	1.0	1.0	24.87	5006451.3	No
Grad Rej	8.02	0.393	0.69001	3.291	64402.6	Yes
Grad (Trig)	9.331	0.338	0.69010	3.346	64716.3	Yes
Grad (Pol)	12.433	0.254	0.69012	3.283	64401.4	Yes
Area Rej	7.286	0.433	0.76088	3.295	63825.3	Yes
Area (Pol)	6.285	0.502	0.76093	3.317	63805.3	Yes
Area (Merc)	17.333	0.182	0.21846	3.39	73010.7	No
Ray Method	14.121	0.223	0.69002	3.318	64134.3	Yes

**Table 3:** Results of using various ellipsoid surface samplers to generate $10^8$ points on a triaxial ellipsoid, using two different random number generators. Each column shows (from left to right): algorithm name, run-time (in seconds), speed relative to naive rejection, acceptance rate, relative standard deviation (as a percentage of the mean), the $\chi ^2$ statistic and whether $\chi ^2$ is smaller than the critical value, $\chi _\textrm{crit}^2 = 65\,033.6$

**Triaxial Ellipsoid**: $(a,b,c) = (3,2,1)$						
RNG	**Lagged Fibonacci** (lagfib4xor)					
**Algorithm**	$t_\textrm{run}$ (s)	speed	*r*	RSD (%)	$\chi ^2$	$\chi ^2 < \chi _\textrm{crit}^2$
Naive Scale	1.488	1.0	1.0	33.063	8716777.8	No
Grad Rej	4.669	0.319	0.64832	3.222	63735.1	Yes
Grad (Trig)	6.237	0.239	0.64838	3.263	64731.0	Yes
Grad (Pol)	9.692	0.154	0.64830	3.27	64894.7	Yes
Area Rej	5.956	0.25	0.71481	3.252	64109.0	Yes
Area (Pol)	5.281	0.282	0.71489	3.234	64490.2	Yes
Area (Merc)	15.64	0.095	0.20620	3.24	65870.9	No
Ray Method	14.205	0.105	0.43220	3.269	64564.7	Yes

RNG	**YARN5s** (yarn5s)					
**Algorithm**	$t_\textrm{run}$ (s)	speed	*r*	RSD (%)	$\chi ^2$	$\chi ^2 < \chi _\textrm{crit}^2$
Naive Scale	3.137	1.0	1.0	33.087	8724015.4	No
Grad Rej	8.592	0.365	0.64833	3.29	64737.6	Yes
Grad (Trig)	10.03	0.313	0.64832	3.234	64219.2	Yes
Grad (Pol)	13.074	0.24	0.64830	3.276	64459.8	Yes
Area Rej	8.521	0.368	0.71483	3.243	64377.8	Yes
Area (Pol)	8.217	0.382	0.71489	3.261	64086.4	Yes
Area (Merc)	28.426	0.11	0.20619	3.227	65387.4	No
Ray Method	21.785	0.144	0.43221	3.276	64486.1	Yes

Tables 2 and 3 show the results for samples of $N=10^8$ random points on an oblate spheroid, (*a*, *b*, *c*) = (3, 3, 1.5), and a triaxial ellipsoid, (*a*, *b*, *c*) = (3, 2, 1), respectively. First consider the uniformity of each method. In both tables, it is immediately clear that the relative standard deviation and $\chi ^2$ statistics from the naive method are much greater than those for all the other algorithms. This is to be expected, since the naive method yields a non-uniform distribution.

Another algorithm that fails the $\chi ^2$ tests is the Mercator coordinate area rejection method. The fact that a uniformity test fails might not be considered surprising, since the method uses a finite truncation on the range of the *v* coordinate, meaning that it is not completely uniform. The loss of uniformity thus occurs near the *c*-axis poles, with fewer generated points than expected in those regions. For every other algorithm, the $\chi ^2$ values are below critical and the relative standard deviations are roughly $3.2\%$.

Looking at the run-times in Tables 2 and 3, the fastest algorithm is the naive method and the slowest is Mercator coordinate area rejection. The former is fast as it simply scales a point generated on the sphere by Marsaglia’s method, while the latter is slow because of a low acceptance rate, combined with the need for trigonometric and hyperbolic function calls. Regardless of efficiency, neither sampler is a good choice due to their non-uniformity.

Of the uniform ellipsoid samplers, the ray intersection algorithm is the slowest. This can be explained in Table 3 by a low acceptance rate. In the oblate case, the value of *r* is higher and the run-time shorter, but the gradient and area rejection methods are still faster. This is because of the algorithmic complexity of the method, with trigonometric function evaluations and multiple random number generator calls.

If one wishes to output the generated point in polar coordinates, the area rejection method is faster than the gradient rejection method for both shape examples. The computational cost of converting from Cartesian to polar coordinates in the gradient rejection method is higher than that of using area rejection and returning polar coordinates directly.

When the output is given in Cartesian coordinates, the fastest uniform sampler depends on the choice of random number generator. For the lagged Fibonacci algorithm, gradient rejection is fastest. Meanwhile, area rejection with Cartesian output has slightly greater speed when using the YARN generator. Tables 2 and 3 show that area rejection has a slightly greater acceptance rate than gradient rejection. Therefore, it might be expected that area rejection is more efficient. However, gradient rejection does not require trigonometric function calls and so each iteration for a proposed point is swifter. With a fast enough random number generator, this outweighs the lower acceptance rate to give a more efficient method.

It should also be noted that the acceptance rates given in Tables 2 and 3 apply only to the particular aspect ratios studied in this analysis. The acceptance rate is a function of aspect ratio and might be expected to decrease as the shape deviates from a sphere.

## Conclusions

Expressions for the surface area of an ellipsoidal patch (as defined by Fig. 1) were derived (see Eqs. 15, 23, 30 and 31). The triaxial patch area expression can be evaluated using a one-dimensional numerical integration algorithm. The integrand requires evaluation of elliptic integrals, for which efficient numerical methods exist. This expression was used to investigate ellipsoid sampling algorithms that are uniform with respect to surface area. The three methods studied in Sect. 5; gradient rejection, area rejection (based on polar coordinates) and ray intersection were all found to result in uniform distributions. For a fast random number generator, the gradient rejection algorithm was found to be the fastest method for generating Cartesian coordinates, with run-time minimised when used with Marsaglia’s method for sphere sampling. For outputs in polar coordinates (or for both Cartesian and polar), the area rejection algorithm is more efficient. This was found to occur for both a spheroid and a triaxial ellipsoid. If one wishes to generate polar coordinates and speed is essential, then the area rejection is preferable. Otherwise, the gradient rejection method is recommended due to its mathematical and computational simplicity.

## Data Availability

Code used to implement the algorithms as well as generate/analyse the data discussed in this article are openly available in the Nottingham Research Data Management Repository at http://doi.org/10.17639/nott.7302. The code is also available on GitHub at https://github.com/cmarples/ESS.

## Appendix A. Maximum value of the area element

In order to use polar coordinates $(\theta ,\phi )$ for the area rejection method, the maximum of the area element function,

69 $$\begin{aligned} s(\theta , \phi ) = \sin \theta \, r(\theta ,\phi ) \, , \end{aligned}$$

where,

70 $$\begin{aligned} r(\theta ,\phi ) = \sqrt{ b^2c^2 \sin ^2\theta \cos ^2\phi + a^2c^2 \sin ^2\theta \sin ^2\phi + a^2b^2 \cos ^2\theta } \, , \end{aligned}$$

must be found. The partial derivatives are given by,

71 $$\begin{aligned} \frac{\partial s}{\partial \theta }&= r(\theta ,\phi ) \cos \theta \left[ 1 + \left( \frac{\sin \theta }{r(\theta ,\phi )}\right) ^2\left( b^2c^2\cos ^2\phi + a^2c^2\sin ^2\phi - a^2b^2 \right) \right] \, , \end{aligned}$$

72 $$\begin{aligned} \frac{\partial s}{\partial \phi }&= \frac{c^2\sin \phi \cos \phi \sin ^2\theta }{r(\theta ,\phi )} \left( a^2 - b^2 \right) \, . \end{aligned}$$

The $\phi$ derivative vanishes if one of the following holds:

$$\begin{aligned} \phi = 0 \mathrm {, \,{\textbf {or}}\,, } \, \phi = \pi /2 \mathrm {, \,{\textbf {or}}\,, } \, \phi = \pi \mathrm {, \,{\textbf {or}}\,, } \, \phi =3\pi /2 \mathrm {, \,{\textbf {or}}\,, } \, \theta = 0 \mathrm {, \,{\textbf {or}}\,, } \, \theta = \pi \mathrm {, \,{\textbf {or}}\,, } \, a = b \,. \end{aligned}$$

For the $\theta$ derivative to vanish, either $\cos \theta = 0$ or the following expression holds,

73 $$\begin{aligned} \left( \frac{\sin \theta }{r(\theta ,\phi )}\right) ^2\left( b^2c^2\cos ^2\phi + a^2c^2\sin ^2\phi - a^2b^2 \right) = -1 \, . \end{aligned}$$

Thus, maxima of the area element satisfy one of the following,

$$\begin{aligned} \theta = \pi /2 \mathrm {, \,{\textbf {or}}\,, } \, \mathrm {Eq. \,\, 73 \,\, holds.} \end{aligned}$$

Both derivatives equal zero when one of the following cases hold:

- Case 1: $\theta = \pi /2$ **and** $\phi = 0$ or $\pi$.
- Case 2: $\theta = \pi /2$ **and** $\phi = \pi /2$ or $3\pi /2$.
- Case 3: $$\theta = \pi /2$$ **and** $$a = b$$.
- Case 4: Eq. 73 holds **and** $\phi = 0$ or $\pi$.
- Case 5: Eq. 73 holds **and** $\phi = \pi /2$ or $3\pi /2$.
- Case 6: Eq. 73 holds **and** $a = b$.
- Case 7: Eq. 73 holds **and** $\theta = 0$ or $\pi$.

Each of the above cases shall now be considered in turn, with the extreme value $s_\mathrm {case \; no.}$ calculated for each. The maximum of these values is then determined.

### Case 1

For $\theta = \pi /2$ and $\phi =0$ or $\pi$, $s(\theta ,\phi )$ reads

74 $$\begin{aligned} s_1 = 1 \times \sqrt{b^ 2c^2 + 0 + 0} = bc \, . \end{aligned}$$

### Case 2

For $\theta = \pi /2$ and $\phi =\pi /1$ or $3\pi /2$, $s(\theta ,\phi )$ reads

75 $$\begin{aligned} s_2 = 1 \times \sqrt{0 + a^2 c^2 + 0} = ac \, . \end{aligned}$$

### Case 3

For a spheroid ($a=b$), *s* does not depend on the $\phi$ coordinate. Thus, at the equator,

76 $$\begin{aligned} s_3 = ac \, . \end{aligned}$$

### Case 4

When $\phi = 0$ or $\pi$, Eq. 73 takes the form,

77 $$\begin{aligned} \sin ^2\theta (b^2 c^2 - a^2 b^2) = - (b^2 c^2 \sin ^2\theta + a^2 b^2 \cos ^2\theta ) \, . \end{aligned}$$

Solving for $\sin ^2\theta$ gives,

78 $$\begin{aligned} \sin ^2\theta = \frac{a^2}{2(a^2 - c^2)} \, , \end{aligned}$$

and so the area element becomes,

79 $$\begin{aligned} s_4 = \sqrt{ \frac{a^2}{2(a^2 - c^2)} \times \left( b^2 c^2 \frac{a^2}{2(a^2 - c^2)} - a^2 b^2 \frac{a^2}{2(a^2 - c^2)} + a^2 b^2 \right) } \, . \end{aligned}$$

### Case 5

For $\phi = \pi /2$ or $3\pi /2$, Eq. 73 reads,

80 $$\begin{aligned} \sin ^2\theta (a^2 c^2 - a^2 b^2) = - (a^2 c^2 \sin ^2\theta + a^2 b^2 \cos ^2\theta ) \end{aligned}$$

By a similar argument to case 4, the area element is,

81 $$\begin{aligned} s_5 = \sqrt{ \frac{b^2}{2(b^2 - c^2)} \times \left( a^2 c^2 \frac{b^2}{2(b^2 - c^2)} - a^2 b^2 \frac{b^2}{2(b^2 - c^2)} + a^2 b^2 \right) } \, . \end{aligned}$$

### Case 6

When $a=b$, the $\phi$ terms disappear from Eq. 73. This leads to,

82 $$\begin{aligned} s_6 = \sqrt{ \frac{a^2}{2(a^2 - c^2)} \times \left( \frac{c^2 a^4}{2(a^2 - c^2)} - \frac{a^6}{2(a^2 - c^2)} + a^4 \right) } \, . \end{aligned}$$

### Case 7

For $\theta =0$ or $\pi$, $\sin \theta =0$, making the left-hand side of Eq. 73 vanish. This means that 73 cannot be true, since $0 \ne -1$. Therefore, this case can never hold.

Assuming a triaxial ellipsoid with $a> b > c$, this leaves three possibilities for the maximum; $s_2$, $s_4$ or $s_5$. First compare $s_4$ and $s_5$. On can determine the larger by taking the difference $s_4 - s_5$ and determining whether it is positive or negative. To simplify the calculation, one can calculate $s_4^2 - s_5^2$ instead (as area elements are always non-negative). Using the SymPy library in Python [40], the difference yields,

83 $$\begin{aligned} s_4^2 - s_5^2 = \frac{a^2 b^2 c^2 (b^2 - a^2)}{4(a^2 - c^2)(b^2 - c^2)} \, . \end{aligned}$$

The numerator is negative, since $a > b$. Meanwhile, the denominator is positive since $a>c$ and $b>c$. Therefore, $s_4^2 < s_5^2$ when $a> b > c$. The maximum value is thus either $s_5$ or $s_2 = ac$. Taking the difference $s_5^2 - s_2^2$ (SymPy was again used) gives,

84 $$\begin{aligned} s_5^2 - s_2^2 = \frac{a^2 (b^2 - 2c^2)^2 }{4(b^2 - c^2)} \, . \end{aligned}$$

The numerator is non-negative and if $b>c$, the denominator is positive. Therefore, $s_5 >= s_2$ for a triaxial ellipsoid and so $s_\textrm{max} = s_5$. For an oblate spheroid with $a = b > c$, the same argument holds and $s_5$ gives the maximum. This leaves two special cases to consider; the prolate spheroid with $a = b < c$, and the sphere.

For a prolate spheroid, $s_4 = s_5$ and the denominator of Eq. 84 is negative. Thus $s_\textrm{max} = s_2 = ac$ is the maximum. For the sphere, the area element is simply $a^2\sin \theta$, so the maximum is $s_\textrm{max} = a^2$.

In summary, the ellipsoid (as defined in Sect. 1) has maximum area element,

85 $$\begin{aligned} \boxed { s_\textrm{max} = {\left\{ \begin{array}{ll} \beta \left( a^2c^2\beta - a^2b^2\beta + a^2b^2 \right) , &{} \text {if triaxial or oblate} \\ ac, &{} \text {otherwise} \\ \end{array}\right. } } \end{aligned}$$

where

86 $$\begin{aligned} \beta = \frac{b^2}{2(b^2-c^2)} \, . \end{aligned}$$

## Footnotes

1 See https://www.numbercrunch.de/trng/
